# Lightweight Detection Methods for Insulator Self-Explosion Defects

**DOI:** 10.3390/s24010290

**Published:** 2024-01-03

**Authors:** Yanping Chen, Chong Deng, Qiang Sun, Zhize Wu, Le Zou, Guanhong Zhang, Wenbo Li

**Affiliations:** 1 School of Artificial Intelligence and Big Data, Hefei University, Hefei 230601, China; 2 Institute of Intelligent Machines, Chinese Academy of Sciences, Hefei 230001, China

**Keywords:** target detection, lightweight, self-explosion defects in insulators, EfficientNet, small target defects

## Abstract

The accurate and efficient detection of defective insulators is an essential prerequisite for ensuring the safety of the power grid in the new generation of intelligent electrical system inspections. Currently, traditional object detection algorithms for detecting defective insulators in images face issues such as excessive parameter size, low accuracy, and slow detection speed. To address the aforementioned issues, this article proposes an insulator defect detection model based on the lightweight Faster R-CNN (Faster Region-based Convolutional Network) model (Faster R-CNN-tiny). First, the Faster R-CNN model’s backbone network is turned into a lightweight version of it by substituting EfficientNet for ResNet (Residual Network), greatly decreasing the model parameters while increasing its detection accuracy. The second step is to employ a feature pyramid to build feature maps with various resolutions for feature fusion, which enables the detection of objects at various scales. In addition, replacing ordinary convolutions in the network model with more efficient depth-wise separable convolutions increases detection speed while slightly reducing network detection accuracy. Transfer learning is introduced, and a training method involving freezing and unfreezing the model is employed to enhance the network’s ability to detect small target defects. The proposed model is validated using the insulator self-exploding defect dataset. The experimental results show that Faster R-CNN-tiny significantly outperforms the Faster R-CNN (ResNet) model in terms of mean average precision (mAP), frames per second (FPS), and number of parameters.

## 1. Introduction

Insulators, as important components of high-voltage transmission lines, serve the functions of electrical separation and support for conductors [1]. Due to their long-term outdoor exposure to sunlight, rain, climate changes, and chemical corrosion, insulators often suffer from self-exploding defects, causing the disconnection of insulator strings and interfering with their performance, thus affecting the safety and stability of power systems [2]. Insulator detection methods are generally divided into two types. The first is manual inspection, where workers directly observe insulators to identify defective parts. However, this method is time-consuming and not safe. The second is intelligent inspection, which can effectively locate defective parts by carrying edge detection equipment on drones for regular inspection of insulators. This is also the current mainstream inspection method.

Currently, the implementation of insulator defect detection mainly relies on traditional methods and deep learning methods. Traditional detection methods primarily differentiate insulators from the background based on features such as size, texture, and color of the images [3]. For example, Tan et al. [4] takes a fusion algorithm based on insulator contour features and grayscale similarity matching. It can extract the contours of insulator pieces, accurately separate them, and construct a defect detection model based on the spacing between insulator pieces and grayscale similarity matching. Liu et al. [5] proposed an edge-based segmentation method for insulator strings. It uses a multi-scale morphological gradient algorithm to extract the edges of insulator strings, determine the largest connected region, and provide guidance for addressing the problem of mis-segmentation of iron caps and umbrella discs caused by edge loss in infrared images of insulator strings. However, these traditional detection methods have low efficiency in feature extraction, poor generalization capabilities, and difficulty in recognizing small-scale and high-likelihood objects in images [6].

To enhance the feature extraction capability and anti-interference ability of insulator detection, traditional detection methods are no longer able to meet modern needs. Many scholars have turned their attention to deep learning methods. For example, Guo et al. [7] used a lightweight target detection network called CenterNet-GhostNet to address the issue of the large number of parameters in the insulator defect detection model, which makes it difficult for unmanned aerial vehicles to deploy on the edge. This network significantly reduces the number of network parameters while achieving a slight increase in detection accuracy, thereby improving the detection speed of the network. Jia et al. [8] considered a lightweight detection method called MDD-YOLOv3. The improved YOLOv3 can quickly and accurately recognize and locate insulator defects in complex backgrounds. Li et al. [9] proposed a method that utilizes multiple-scale feature encoding and dual attention fusion to improve the accuracy and speed of detecting insulator defects in transmission lines. It has a certain reference value for accurate insulator defect detection by unmanned aerial vehicles. In summary, compared with the traditional manual feature extraction of insulators, deep learning-based detection methods can automatically and accurately extract target features and have stronger generalization capabilities.

In recent years, due to the development of “Intelligentization” in the power system, the combination of using drones to collect insulator defect data and computer vision technology has become a popular method for intelligent inspection [10,11]. However, deep learning-based object detection networks usually require a large number of computational resources and parameters for training and inference, which limits their deployment and usage in practical applications. Therefore, the construction of lightweight detection models has become crucial [12,13,14].

The existing deep learning detection methods can be mainly divided into two categories. One is the two-stage detection model represented by R-CNN, Faster R-CNN, and Mask R-CNN. These algorithms require two-stage processing: (1) candidate region acquisition and (2) classification and regression of candidate regions. The other is the single-stage detection model represented by the YOLO series, which simultaneously obtains candidate regions and categories through joint decoding. Among them, the Faster R-CNN model, as a representative of two-stage networks, exhibits a more pronounced advantage when it comes to handling high-precision, multi-scale, and small object detection tasks. However, the original Faster R-CNN (ResNet) model suffers from significant drawbacks in terms of detection speed performance. Firstly, its feature extraction capability is relatively poor. This is because the original ResNet cannot effectively extract high-level semantic information and low-level fine-grained features from images, making it difficult for deeper feature maps to learn information about small objects. Secondly, the network’s inference speed is slow. The original model contains a lot of redundant information, resulting in a slow detection speed. Finally, the network parameters are not well optimized. For instance, the original model’s learning rate can easily get stuck in local optima, leading to a decline in the overall model performance.

In this paper, we have lightweighted the original Faster R-CNN (ResNet) and constructed a new detection model (Faster R-CNN-tiny), as shown below:

(1) We use the lightweight EfficientNet [15] as the backbone network to capture multi-scale detailed features of faulty object. These features serve as inputs to the Feature Pyramid Network (FPN) [16], enhancing the network’s capability to extract characteristics from defects of various scales.
(2) A feature fusion module is added to effectively combine high-level semantic information with low-level detail information, enhancing the accuracy of defect detection. Ordinary convolutions in the network are replaced with depth-wise separable convolutions (DSConv), which improve the detection speed to some extent.
(3) Transfer learning methods [17] are employed in the network training process, combining freezing and unfreezing training strategies to enhance the network’s detection performance for defects in complex environments.
(4) The proposed lightweight Faster R-CNN-tiny object detection model can effectively locate the defects of insulators by learning a large number of features from self-made defect images of insulators. This is a crucial step towards the edge detection of defects in the next step. We have also introduced a new dataset for insulator defects called Tiny-Insulator.

The rest of this paper is structured as follows: In Section 2, we introduce the structure and principles of the original Faster R-CNN model. In Section 3, we provide a detailed description of the target model, Faster R-CNN-tiny, analyzing the functionality and flow of each component. In Section 4, we validate the impact of different network structures on experimental performance using the insulator defect dataset. In Section 5, we summarize this research and discuss future work.

## 2. Related Works

### 2.1. Faster R-CNN

Defect detection involves the following two tasks: defect classification and localization. This paper chooses two-stage Faster R-CNN [18] as the lightweight base network structure, which exhibits a high accuracy in object detection tasks. Its working principle is to first identify and locate defective insulators in an image, then select them with rectangles, and, finally, mark their belonging categories near the rectangles.

**Figure 1:**
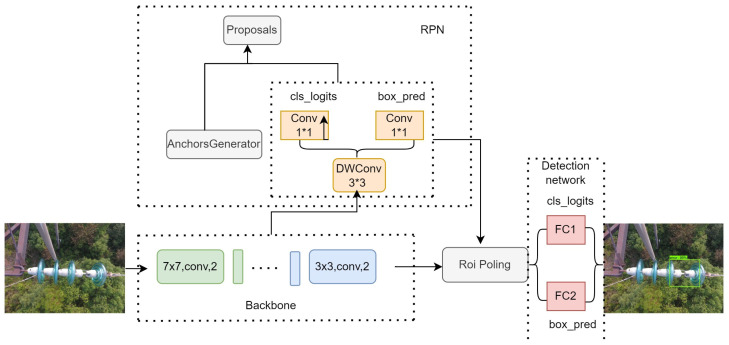
The original Faster R-CNN network structure.

Faster R-CNN is a two-stage object detection network proposed by Ross B. Girshick, building upon the foundations of R-CNN and Fast R-CNN. As shown in Figure 1, the Faster R-CNN network structure consists of four parts: the backbone network, the Region Proposal Network (RPN), the Region of Interest (RoI) pooling, and the detection network. The backbone network is a ResNet network stacked with multiple 7 × 7 convolutions of stride 2 and 3 × 3 convolutions of stride 2. The RPN is a feature-processing part composed of two parallel 1 × 1 convolutions by 3 × 3 deep separable convolutions (DWConv). The detection network consists of two parallel fully connected layers (FC).

The entire algorithm process is divided into several parts. First, the backbone network extracts features from preprocessed images by capturing multi-scale information with inter-channel interactions. Then, these features are used as input for the RPN, which generates candidate boxes. The candidate boxes are mapped to the feature map output by the backbone network. The obtained feature matrix is passed through the RoI Pooling layer, resulting in a 7 × 7 feature map. Finally, the detection network utilizes the feature map to obtain class information and bounding box regression parameters. The candidate boxes are adjusted using the bounding box regression parameters to obtain the final target position.

To address the low accuracy and slow speed issues of the original model in insulator defect detection, we propose a lightweight defect detection model based on Faster R-CNN-tiny. The aim is to make the original detection model more suitable for future edge deployment requirements.

### 2.2. ResNet

ResNet, which stands for Deep Residual Network, is a landmark convolutional neural network (CNN) that uniquely solves the problems of gradient disappearance and explosion in deep neural networks.

In 2015, ResNet won the ILSVRC (ImageNet Large Scale Visual Recognition Challenge) championship and significantly improved error accuracy in the ImageNet classification task. This is mainly due to ResNet’s “shortcut connections”, also known as “skip connections”. Through this connection method, the output of the deep network can be directly added to some layers of the shallow network, which helps the gradient to be directly transmitted to the shallow network. This design allows the network to train deep networks with dozens or even hundreds of layers.

### 2.3. EfficientNet

**Figure 2:**
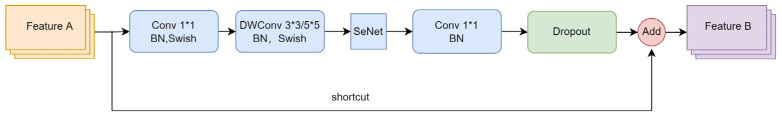
The MBConv module in the EfficientNet network.

EfficientNet, proposed by Google in 2019, constructs models through compound scaling to improve model efficiency. It is composed of one ordinary convolutional layer and sixteen mobile inverted bottleneck convolution modules (MBConv). Among them, the MBConv module is its core component, which mainly draws inspiration from the residual structure of MobileNetv3 [19]. As shown in Figure 2, it has the following functional features: firstly, a Swish activation function [20] is used instead of a ReLU activation function, and Swish performs better on deep models. Secondly, a squeeze-and-excitation networks (SENet) [21] attention mechanism is added to each MBConv module to strengthen the extraction of small-scale target features and suppress useless feature information. Thirdly, dropout layers are introduced. When there are shortcut branches (shortcuts), the main branch of the whole module will be randomly dropped, leaving only the shortcut branch, making the network lighter and improving the detection speed of the model.

The main difference between the two lies in their network structure and optimization strategies. EfficientNet adopts a deeper and wider network structure, while using compound scaling to adjust the depth, width, and resolution of the network. This makes EfficientNet reduce the number of parameters and computations, thereby improving the efficiency of the model. On the other hand, ResNet mainly solves the vanishing and exploding gradient problems using residual blocks, with a relatively simple network structure.

## 3. Methodology

**Figure 3:**
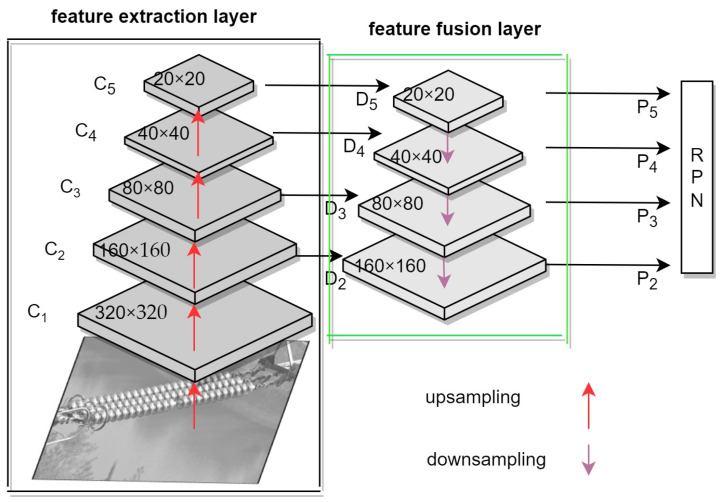
The backbone of the Faster R-CNN-tiny model.

In order to make the model more suitable for the detection of small targets and reduce the number of model parameters, this paper proposes a new object detection model called Faster R-CNN-tiny. The Faster R-CNN-tiny model only improves the backbone part of the original Faster R-CNN model, as shown in Figure 3. The input image first goes through a feature extraction layer (EfficientNetB0) to obtain feature maps at different resolutions (C2, C3, C4, C5), then enters a feature fusion layer (Feature Pyramid Network), and, finally, the resulting different feature maps (P2, P3, P4, P5) are further processed in the RPN.

To enable Faster R-CNN-tiny to detect more small object features, we have added a D2 object detection layer to detect shallower features. This can be specified as follows: First, the use of EfficientNet with attention mechanisms as the backbone network for feature extraction from input images, addressing the issue of partial feature information loss in the generation of multi-resolution feature maps by the backbone network [22]. After that, a lightweight feature fusion module is proposed and added to the backbone network. This module effectively integrates low-level positional information with high-level semantic information, ensuring that the fused feature maps retain sufficient detailed information. Finally, DSConv are employed to replace regular convolutions in the FPN and RPN. This not only reduces the network’s parameter count but also enhances its detection speed.

To ensure that the target detection algorithm has a good scale invariance, the original Faster-RCNN algorithm generates anchor boxes with ratios of (1:1, 1:2, 2:1) and sizes of (64, 128, 256) when traversing the feature map. However, the original Faster-RCNN algorithm’s anchor boxes are not suitable for detecting small targets or actual-scale defective insulator targets. To obtain better anchor box ratios, this paper statistically analyzes the length-to-width ratios of the defective insulators in the dataset. The length-to-width ratio of the insulators is approximately 60% for 1:1, 26% for 2:1, 11% for 3:1, and 3% for 4:1. Therefore, in this paper, the anchor box ratios are set as (1:1, 2:1, 3:1) with sizes of (16, 32, 64, 128, 256).

After a series of consecutive convolutional and pooling operations on the input image, the information on the feature map gradually diminishes. In Figure 3, the C2 feature map layer contains more object information than the C3 feature map layer. Therefore, this paper introduces detection in the C2 feature layer, which contains more feature information. In the original Faster R-CNN algorithm, the feature extraction part only utilized ResNet, whereas the new model incorporates EfficientNet and FPN. When the input image size is 640 × 640, the detection layer corresponding to C3 has a size of 80 × 80, suitable for detecting objects larger than 8 × 8; the detection layer corresponding to C4 has a size of 40 × 40, suitable for detecting objects larger than 16 × 16, and the detection layer corresponding to C5 has a size of 20 × 20, suitable for detecting objects larger than 32 × 32.

### 3.1. Feature Extraction Layer

**Figure 4:**
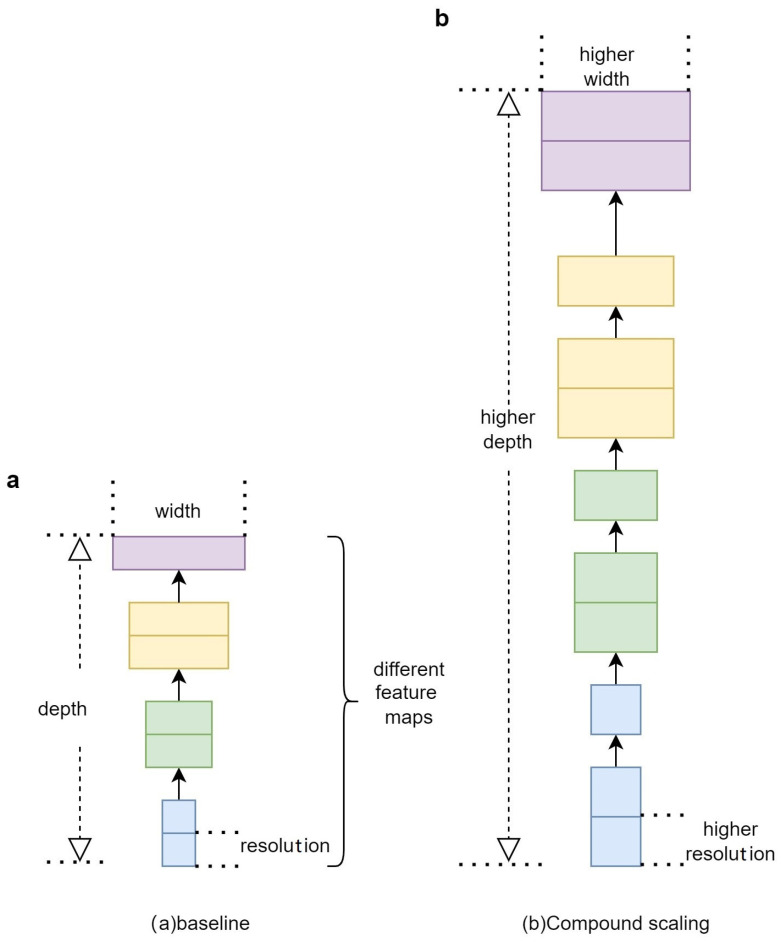
Illustrates the NAS search process within EfficientNet. (**a**) baseline and (**b**) compound scaling.

Traditional object detection algorithms have many similarities between the feature layers in their feature extraction networks. While these similar feature layers improve accuracy, they also introduce a lot of redundant information, making the network models large and difficult to deploy on small mobile devices [23,24]. Therefore, research on lightweight networks has become a hot topic. Currently, the mainstream lightweight networks include SqueezeNet, ShuffleNet, EfficientNet, RegNet, and MobileNet. Considering that the detection targets are small objects and that the purpose is to build a lightweight and efficient feature extraction network, EfficientNetB0 is chosen as the backbone network. EfficientNet is a network model obtained through Neural Architecture Search (NAS), and it achieves EfficientNet B0~B7 network models by rationalizing the configuration of the following three parameters: image input resolution (r), network depth (d), and network width (w), as shown in Figure 4.

Then, the NAS search is used to obtain the best *r*, *d*, and *w* factors for the EfficientNet network.

(1) s.t:N(d,w,r)=F^i=1…s⊙id·L^i(X〈r·H^i,r·W^i,w·C^i〉)

(2) [Accuracy(N(d,w,r))]d,w,rMAX

(3) Memory(N)≤Target_memory

(4) FLOPs(N)≤Target_flops

In Equations (1)–(4), $\begin{array}{c} ⊙\\ i=1\ldots s \end{array}$ represents the multiplication operation. ${\hat{F}}_{i}^{d\cdot {\hat{L}}_{i}}$ represents that the ${\hat{F}}_{i}$ operation is repeated $d\cdot {\hat{L}}_{i}$ times in the i-th stage. ${\hat{F}}_{i}$ represents an operation. Here, $X_{<r\cdot {\hat{H}}_{i},r\cdot {\hat{W}}_{i},w\cdot {\hat{C}}_{i}>}$ represents the feature matrix of the *i*-th stage’s input; $<{\hat{H}}_{i},{\hat{W}}_{i},{\hat{C}}_{i}>$ represents the height, width, and number of channels of *X*. Moreover, *d*, *r*, and *w* are used for scaling, respectively, ${\hat{L}}_{i},\;{\hat{H}}_{i}$, and ${\hat{C}}_{i}$. Memory and FLOPs represent the limitations of the hardware’s memory and maximum computational load.

By utilizing the MBConv module mentioned above (Figure 2), a lightweight network structure called EfficientNet can be constructed. Depending on the network’s different stages, the MBConv structure can be modified in various ways. Firstly, the number of channels in the input features is expanded through 1 × 1 convolutions. Depending on the needs of each stage, DSConv can be selected in either a 3 × 3 or 5 × 5 size to integrate the extracted features and reduce noise. Then, SENet modules are used to enhance the ability to extract features at small target scales, followed by dimensionality reduction using 1 × 1 convolutions. Finally, shortcut connections only exist when the shape of the input feature matrix and the final output feature matrix are the same, allowing for the superposition of two types of features to enhance the network’s feature extraction capabilities.

### 3.2. Feature Fusion Layer

The original Faster-RCNN object detection network only uses a single feature layer with high-order semantic information to predict target information. For small object detection, the high-order semantic feature maps lack the underlying information about details and have an impact on the accuracy of the detection results [25,26]. The new model leverages the multi-resolution feature maps from the feature extraction layer; these are merged through the feature fusion layer to retain the panoramic information on the targets.

**Figure 5:**
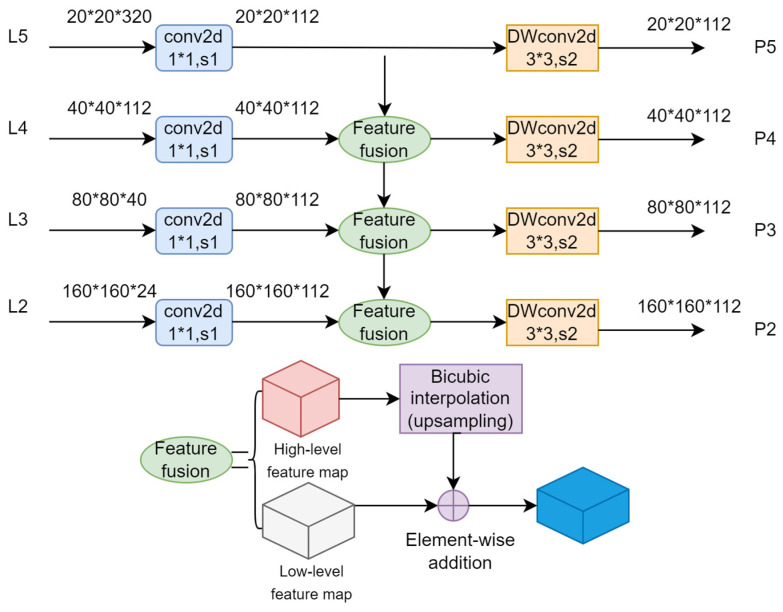
Feature fusion layer construction.

To provide a more intuitive understanding of the principle behind the feature fusion layer, we have separately illustrated the feature fusion layer in Figure 5, as shown. Different resolution feature maps of 20 × 20, 40 × 40, 80 × 80, and 160 × 160 obtained from the feature extraction layer serve as inputs to the feature fusion layer. These four feature maps of varying scales contain rich semantic and positional information, and their effective fusion results in the final full-sized feature map, greatly enhancing the network’s detection performance. Firstly, a step-1 1 × 1 convolution operation is applied to the four feature layers—L2, L3, L4, and L5—obtained from the feature extraction layer, resulting in dimensions of 112 and providing the necessary conditions for the subsequent feature fusion. Then, the higher-level feature maps are upsampled using bicubic interpolation and are pixel-wise added to the corresponding lower-level feature maps from the next layer to produce the initial feature fusion map. Finally, a DSConv operation is performed on the initial feature fusion map to integrate the extracted features and reduce noise interference on the detection results. 

### 3.3. Depth-Wise Separable Convolution

Addressing the issues of large network size and slow detection speed, a lightweight network structure is proposed. The common convolutions in the FPN and RPN are replaced with DSConv [27], which reduces network parameters and computation while increasing detection speed.

**Figure 6:**
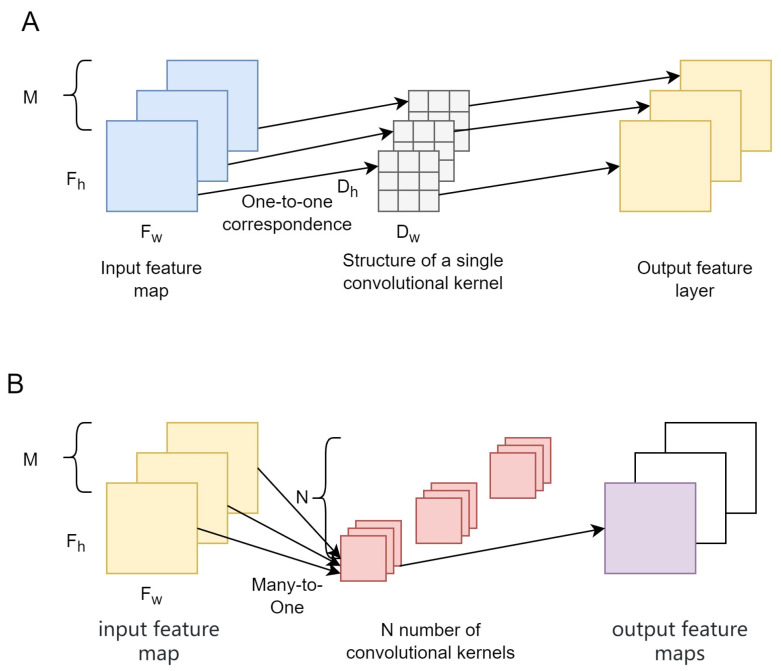
Structure of DSConv: (**A**) the process of DWConv and (**B**) the process of PoConv.

As shown in Figure 6, DSConv consists of two main steps: channel-wise convolution (DWConv) and point-wise convolution (PoConv). DWConv produces feature maps with the same number of channels as the input by applying a convolutional kernel to each channel in the feature map after the convolution procedure. PoConv establishes dimensional links between the feature maps created by DWConv by further convolving them using a 1 × 1 convolution.

The parameter volume of a normal convolution is $D_{w}\times D_{h}\times M\times N$, while that of a channel−wise convolution is $D_{w}\times D_{h}\times M\times 1$ and that of point−wise convolution is $D_{w}\times D_{h}\times 1\times 1$. The ratio of the parameter volume of DSConv to that of a normal convolution is the following:

(5) $$\;\;\;\;\;\frac{D_{w}\times D_{h}\times M\times 1+1\times 1\times M\times N}{D_{w}\times D_{h}\times M\times N}=\frac{1}{N}+\frac{1}{D_{w}\times D_{h}}\;$$

For feature maps in which the scale does not change after processing, the calculation volume of a normal convolution is $D_{w}\times D_{h}\times F_{w}\times F_{h}\times M\times N$. The calculation volume of DWConv is $D_{w}\times D_{h}\times F_{w}\times F_{h}\times M\times 1$ and that of PoConv is $1\times 1\times F_{w}\times F_{h}\times M\times N$. The ratio of the calculation volume of DSConv to that of a normal convolution is as follows:

(6) $$\;\;\;\;\;\;\;\frac{D_{w}\times D_{h}\times F_{w}\times F_{h}\times M\times 1+1\times 1\times F_{w}\times F_{h}\times M\times N}{D_{w}\times D_{h}\times F_{w}\times F_{h}\times M\times N}=\frac{1}{N}+\frac{1}{D_{w}\times D_{h}}$$

Among them, $D_{w}\;\mathrm{and}\;D_{h}$ represent the width and height of the convolution kernel; M represents the dimension of the input feature map; N represents the number of convolutional kernels; $F_{w}$ and $F_{h}$ represent the width and height of the feature map.

Due to the fact that, in normal circumstances, the number of convolutional kernels (N) is much greater than the size of the convolution kernel $D_{w}\times D_{h}$, when the depth-separable convolution kernel size is 3 × 3 × M, Equations (1) and (2) are approximately equal to 1/9. Therefore, it can be seen that depth-separable convolution greatly reduces the parameter and computation amount of network models, thereby improving the detection speed of the network models.

### 3.4. Training Network Models with Transfer Learning

ImageNet [28] is an authoritative benchmark for evaluating network performance, with more than 1.2 million images which are finely classified into 1000 categories. In addition, ImageNet provides a large number of pre-trained weights that can be used in current object detection tasks, while revealing key features such as edges, corners, textures, and other characteristics in natural images, laying the foundation for visual tasks.

Due to safety concerns in power systems, it is difficult for drones to obtain data on defective insulators in transmission lines, and using small datasets to train models can easily lead to the slow or non-convergence of networks, resulting in poor detection performance [29,30]. To improve the detection performance of networks, transfer learning methods are introduced into the model training process, combining common feature knowledge of objects with the target object, thereby improving the detection performance of the target object.

This paper first uses the ImageNet dataset to pre-train the main network EfficientNet and obtain new model weights for the main network. Then, in order to save hardware costs, a combined model training strategy of freezing and unfreezing is used, using a homemade small dataset of insulator defects to re-fine-tune the network so that the network model can quickly adapt to small sample insulator defect datasets [31].

## 4. Experimental Results and Additional Requirements of the Analysis

### 4.1. Experimental Setup and Evaluation Metrics

The software environment used in all the experiments in this paper includes Python 3.6, Pycharm Community Edition 2022.2.1, and the Pytorch (1.10.2) deep learning framework. The CPU model is an Intel Core i7-12700H @ 2.30 GHz; the GPU model is an NVIDIA GeForce RTX 3070 with 8GB of memory and 16 GB of RAM, and the experiments were conducted on a PC. The detection performance of the model on self-exploding insulators was measured using the following three evaluation metrics: mean average precision (AP), frames per second (FPS), and parameters (Para).

### 4.2. Dataset

**Figure 7:**
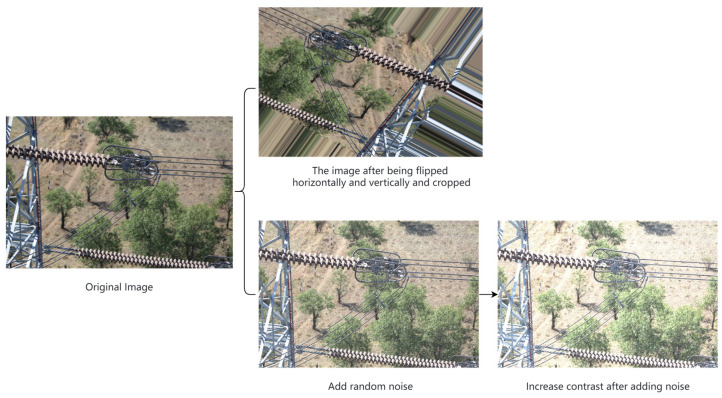
Data augmentation.

Since there is currently no publicly available insulator defect dataset, this paper proposes a new Tiny-Insulator dataset consisting of 400 insulator defect images and 400 annotation files. The initial dataset is divided into a 3:1 ratio for obtaining training and validation sets, with the training set being used for augmentation and network training and the validation set being used to evaluate network performance. These images are primarily sourced from a well-known power station in the region. To enhance the model’s generalization and prevent overfitting, data augmentation techniques are applied to the training dataset, including the addition of noise and image transformations like flips. Brightness and contrast adjustments are also employed to simulate various insulator environments, thereby improving the robustness of model training. As depicted in Figure 7, the final training dataset is augmented to include 600 images and 600 annotation files.

To address the significant differences in resolution among the self-made insulator images, all images were resized to 640 × 640 pixels to create the final dataset. The defects on the insulators were labeled using the Labelimg (1.8.6) image annotation software, and all label files were organized in the PASCAL VOC dataset format.

### 4.3. Network Training

**Figure 8:**
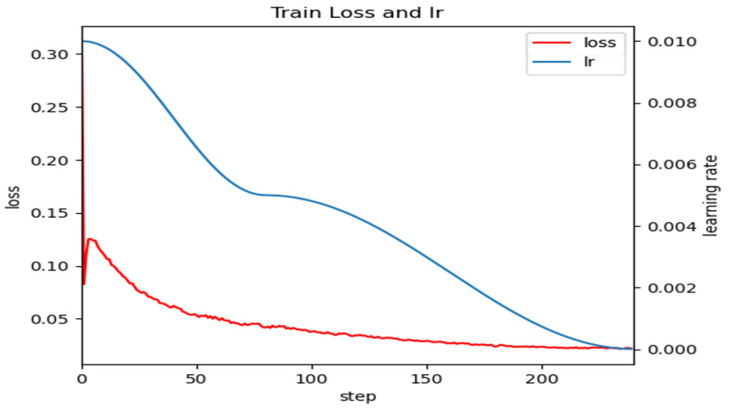
Change in training loss and learning rate curve.

The batch size was set to four, and Adam was selected as the optimizer. The number of iterations for model training was 300, measured in epochs, and there were two categories (one background). In order to prevent the neural network model from getting stuck in local optima, a cosine annealing learning rate decay is used, with an initial learning rate set to 10 × 10^−2^ and a decay multiplier factor of 10 × 10^−3^. To avoid a drop in model performance when training on a self-made small dataset, a transfer learning approach is employed. First, the backbone network is pretrained on a large-scale dataset like ImageNet, and its pretrained weights are used as the initial weights for the entire network. The model is trained using a combination of frozen and unfrozen layers to prevent issues arising from large discrepancies between the initial network weights and the actual data distribution. This approach also saves hardware resources and speeds up network convergence. During training, the weights of the backbone network are frozen for the first 30 epochs, and the remaining network structure is fine-tuned. The remaining 210 epochs involve unfreezing and training the entire network, resulting in increased GPU memory usage and changes to all model parameters. The loss and learning rate change process of the lightweight network model is shown in Figure 8.

As shown in Figure 8, transfer learning can significantly improve the convergence speed of the network.

There are three stages in the change in model loss. Firstly, the loss value decreases rapidly during the first 50 steps. Then, when training is between 50 and 150 steps, the loss decreases slowly. Finally, after the training iterations reach 150 steps, the loss gradually tends to be stable around 0.015. The learning rate decreases gradually along with the increase in iteration steps and, eventually, drops to a level close to the loss value.

### 4.4. Performance Analysis of Faster R-CNN-Tiny Models

#### 4.4.1. Main Network Selection

According to the evaluation results generated using the Faster R-CNN target detection framework and by combining it with five different lightweight backbone networks in Table 1, the following points can be seen. The mean average precision (mAP) metric using EfficientNetB0 as the backbone network reaches 85.1%, which is 22.7% higher than ShuffleNetV2, 9.6% higher than SqueezeNet, 6.2% higher than MobileNetV3, and 5.6% higher than RegNetY800MF. In addition, the parameter count (Params) of EfficientNetB0 as the backbone network is lower or equal to that of the other four backbone networks, which is only 5.3M.

These results show that EfficientNetB0 not only has stronger feature extraction capabilities for defective insulators but also requires fewer parameters. Therefore, this research selects EfficientNetB0 as the feature extraction network.

#### 4.4.2. Ablation Experiment

To verify the effectiveness of the proposed lightweight network structure, we conducted an ablation experiment in the same experimental environment to evaluate the impact of the improved modules on target detection. We chose the original Faster R-CNN with ResNet50 as the backbone network and trained it for 240 epochs. The results are shown in Table 2. The main evaluation metrics used were mAP_0.5_ (which measures the target detection accuracy at an IoU threshold of 0.5), Params (which represents the size of the network), GFLOPs (which represents the model’s computational complexity), and the symbol “✓” represents the use of this network structure.

As shown in Table 2, the first row shows the original Faster_rcnn algorithm without any detection results from any improved modules. Each subsequent row gradually adds different improved modules. The last row of the table shows the proposed Faster R-CNN-tiny model.

The following conclusions can be drawn. Firstly, when comparing the original target detection network Faster R-CNN with the replaced lightweight backbone network EfficientNetB0, the network parameters are reduced by 62.9 M and GFLOPs by 82.5 G, while the detection accuracy is improved, indicating that replacing the backbone network with EfficientNetB0 significantly improves the detection performance of the network. Secondly, the addition of the feature fusion module (FPN) leads to a significant improvement in model mAP_0.5_ but also results in an increase in parameters, proving that fully integrating high-level semantic information and low-level position information into the model is indeed useful for improving its detection accuracy. Thirdly, replacing ordinary convolutional layers with DSConv makes the model lighter and more complex, resulting in a slight increase in detection speed under slightly lower detection accuracy, demonstrating the effectiveness of DSConv. And, finally, adding new anchor boxes on top of these experiments results in a slight increase in the model’s detection accuracy, as adding small-scale detection anchor boxes better adapts to insulator defect detection in this paper. To sum up, this paper performs lightweight improvements on the Faster R-CNN algorithm, which not only greatly improve the detection accuracy of the model but also ensure real-time detection, demonstrating the effectiveness of the improved modules on model performance.

#### 4.4.3. Detection Result Visualization

**Figure 9:**
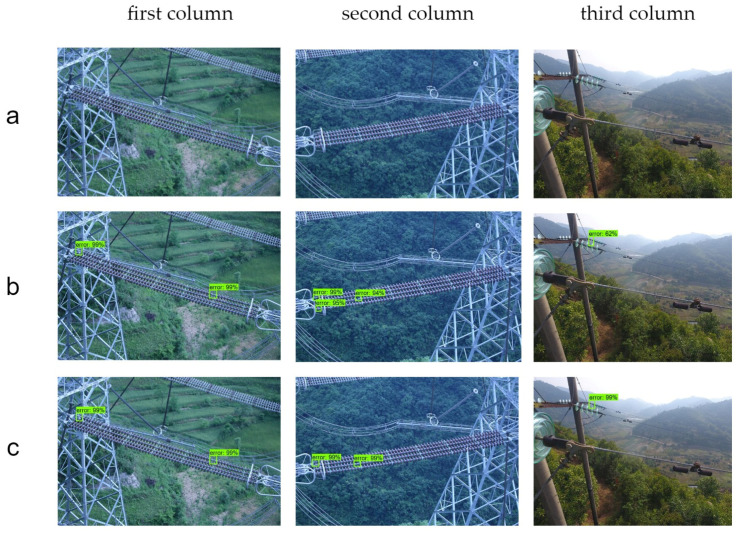
Visualization results of model improvement before and after.

To further verify the detection effectiveness of the improved algorithm on self-exploding insulator defects in practical power inspection, the detection results are visualized and compared. As shown in Figure 9, Line a represents the real inspection chart; Line b represents the original Faster R-CNN detection result, and Line c represents the Faster R-CNN model detection result after the lightweight optimization outlined in this paper.

As can be seen from the visualization results in Figure 9, for the detection of normal small targets, as shown in the first column, both the original Faster R-CNN algorithm and the improved algorithm can be recognized. For the detection of multiple targets in complex environments, as shown in the second column, the original algorithm has false positives, while the improved algorithm can accurately detect them. In the third column, although both algorithms can be recognized, the improved algorithm has a higher confidence. Based on the comprehensive evaluation metrics and image visualization results, the model proposed in this paper can effectively locate defective insulators in different environments.

#### 4.4.4. Comparison Experiment on Different Object Detection Algorithms

To further validate the superiority of the algorithm proposed in this paper, a comparative experiment is conducted on the same hardware environment setup and power transmission line self-destructing insulator dataset with current mainstream object detection algorithms, including Faster R-CNN, RetinaNet, YOLOv5s, and YOLOv6s. The evaluation metrics used in the experiment are the number of parameters (Params), the average precision (AP), and the frames per second (FPS). The FPS metric represents the number of images the detection model can process per second and is commonly used to assess the detection speed of object detection networks. The experimental results are shown in Table 3.

As can be seen from Table 3, with a certain level of detection speed, the detection accuracy of Faster R-CNN-tiny algorithm surpasses the other algorithms. Compared with the original Faster R-CNN and RetinaNet, the average precision (mAP_0.5_) is improved by 7.3% and 12.9%, respectively. The frames per second (FPS) are also increased by 13.4 and 12.9, indicating that this algorithm exhibits high detection efficiency for self-destructing insulator defects. Moreover, the parameter count of this paper’s algorithm is significantly smaller than those of the other two algorithms, indicating a smaller model size. Compared to the YOLO algorithm, although the detection speed of this paper’s algorithm is slower than YOLOv5s and YOLOv6s by 50.0 frames per second and 63.6 frames per second, respectively, it possesses certain advantages in terms of detection accuracy and model size. In summary, this paper’s algorithm enhances network detection accuracy while considering network detection speed and model size, meeting the real-time and efficient detection requirements for defective insulators.

## 5. Conclusions

To address the issues of large volume and slow detection speed of the original Faster R-CNN model, an insulator defect detection model based on Faster R-CNN-tiny is proposed. The experimental results show that, compared with the original algorithm, the AP and FPS of this algorithm have improved by 7.3% and 13.4 frames/second, respectively, and the model parameter has decreased from 70.5 M to 10.4 M. This model can effectively identify insulator defects in transmission lines and has a certain reference significance for maintaining national line safety.

In the next stage of research, we will attempt to combine the new model with an embedded platform to prepare for future edge detection of the model. At the same time, we will also use other datasets in the power grid for testing. This will enable the model to have a better network structure and a higher detection accuracy.

## Figures and Tables

**Table 1:** Experimental results of different backbone networks.

Object Detection Framework	Backbone Network	mAP_0.5_ (%)	Params (M)
	ShuffleNetV2	62.4	5.3
	SqueezeNet	75.5	6.9
Faster R-CNN	MobileNetV3	78.9	5.8
	RegNetY800MF	79.5	6.3
	EfficientNetB0	85.1	5.3

**Table 2:** Evaluation results of ablation experiments.

Network Framework		Improvement			Evaluation Results		
	Feature Processing				mAP_0.5_	Params	GFLOPs
	EfficientNetB0	Feature Fusion	DSConv	New Anchor Box	(%)	(M)	(G)
					83.0	70.5	88.8
	✓				85.1	7.6	6.3
Faster R-CNN	✓	✓			90.4	10.9	19.8
	✓	✓	✓		90.0	10.4	11.8
	✓	✓	✓	✓	90.3	10.4	11.8

**Table 3:** Comparison of different models.

Network Model	Backbone Network	Params (M)	mAP_0.5_ (%)	FPS (F/S)
Faster R-CNN	ResNet50	70.5	83.0	21.8
RetinaNet	ResNet50 + FPN	32.2	86.5	22.3
YOLOv5s	CSPDarknet	7.0	87.3	50.0
YOLOV6s	EfficientRep	17.1	87.7	63.6
Ours	EfficientNetB0	10.4	90.3	35.2

## Data Availability

Data are contained within the article.
